# Hypertrophic Cardiomyopathy Genotype–Phenotype Analysis in Lithuanian Single-Center Cohort

**DOI:** 10.3390/ijms27010221

**Published:** 2025-12-25

**Authors:** Marius Šukys, Eglė Ereminienė, Kristina Aleknavičienė, Rimvydas Jonikas, Eglė Tamulėnaitė-Stuokė, Joana Ažukaitė, Rasa Ugenskienė

**Affiliations:** 1 Department of Genetics and Molecular Medicine, Medical Academy, Lithuanian University of Health Sciences, 50161 Kaunas, Lithuania; marius.sukys@lsmu.lt (M.Š.);; 2 Department of Cardiology, Medical Academy, Lithuanian University of Health Sciences, 50161 Kaunas, Lithuaniaegle.tamulenaite@kaunoklinikos.lt (E.T.-S.); joana.azukaite@kaunoklinikos.lt (J.A.); 3 Institute of Cardiology, Lithuanian University of Health Sciences, 50103 Kaunas, Lithuania

**Keywords:** hypertrophic cardiomyopathy, genetic testing, diagnostic yield

## Abstract

Hypertrophic cardiomyopathies (HCMs) are among the most common genetic disorders; however, they might be underdiagnosed. Sequencing core sarcomere gene panels remain the main diagnostic tool. We present the results of HCM genetic testing performed at Lithuania’s tertiary care center. All patients with diagnosed or clinically suspected HCM underwent next-generation panel sequencing. Of 204 patients, 34 (16.7%) received a genetic diagnosis. The most commonly affected genes were *MYBPC3* and *MYH7*. Notably, two patients were found to have LEOPARD syndrome due to *PTPN11* gene variants. Our results indicate that patients with an identified pathogenic variant were diagnosed with HCM at a younger age and exhibited a more severe phenotype (greater septal wall thickness), although no clear correlation with family history was observed. In addition, four novel *MYBPC3* variants, c.3467dup, c.1503C>G, c.2610dup, and c.1251del, were identified.

## 1. Introduction

Cardiovascular diseases remain the leading cause of death worldwide. In Lithuania, according to the data from the National Hygiene Institute, cardiovascular diseases account for 52.7% of all causes [1]. This number is one of the highest in Europe, as Europe’s average is 32.7% [2]. This highlights the need for a closer look at the underlying structure of these disorders. From a genetic perspective, the largest contribution to disease risk comes from polygenic risk variants. These remain difficult to interpret, as not all of them have been identified; their individual effect sizes are uncertain, and interactions between genetic variants are still poorly understood. Monogenic variants are easier to interpret but are much rarer in the general population.

Possibly the first description in history of HCM was in 1958 by Sir Russell Brock, who presented three cases of pulmonary stenosis due to chronic pressure overload, which manifested as left ventricular outflow tract hypertrophy [3]. In 1961, it was recognized as a distinct disease and was given the name idiopathic hypertrophic subaortic stenosis [4]. Today, the European Society of Cardiology describes HCM as “left ventricular wall thickness ≥ 15 mm in any myocardial segment that is not explained solely by loading conditions” [5]. Many studies have reported a disease frequency of 1 in 500 people. These studies were based on screening large cohorts with heart ultrasound or heart magnetic resonance [6]. A number of other studies looked at health record data and found a much lower frequency, about 1 in 2000. This suggests that many cases are underdiagnosed and may lack appropriate health management.

In 2014, the European Society of Cardiology reported that genetic testing identifies pathogenic variants in sarcomere genes in approximately 40–60% of HCM cases, 5–10% are syndromic cases, and 25–30% remain of unknown origin [7]. An increasing number of genetic diagnoses can be expected as more HCM-related genes are discovered and genetic testing technologies continue to improve. However, in 2020, A. Butters reported a genetic testing yield of about 32% in HCM cases [8]. More recent studies have shown even lower yields: L.M. Dellefave-Castillo reported 22.1% from 2596 cases [9], and J. Hathaway reported 26.8% from 1376 cases [10]. Comparisons between studies may be challenging, as patient selection criteria and variant pathogenicity assessment methods (i.e., which guidelines were used) may differ. Consequently, the true prevalence and genetic landscape of HCM remain difficult to define, and considerable uncertainty persists regarding the underlying causes of many cases. Therefore, in this study, we aim to analyze the genetic profile and clinical characteristics of the most common monogenic heart disorder, hypertrophic cardiomyopathy, in a tertiary university hospital in Lithuania.

## 2. Results

**Figure 1:**
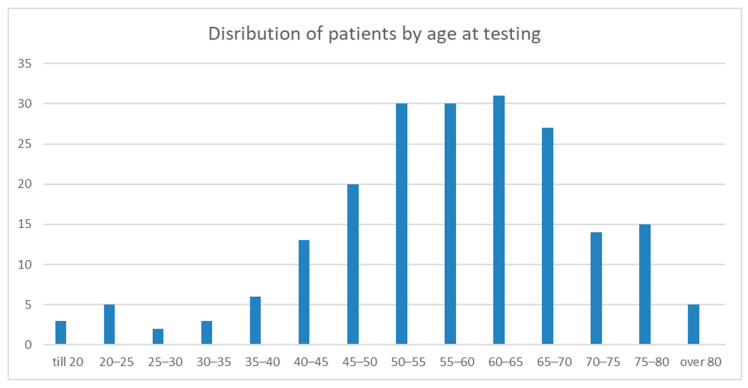
The graph shows patient distribution by age at which they were tested for HCM. The Y-axis shows the age group. The X-axis shows the patient number.

A total of 204 patients underwent genetic testing for HCM. The mean age at testing was 57.49 ± 13.68 years (range 18–86) (Figure 1). The study cohort consisted of 142 males (69.6%) and 62 females (30.4%). The main clinical parameters are presented in Table 1.

When comparing the genders, females were significantly older at the time of testing than males (median age 63.87 vs. 54.72 years, *p* < 0.001, Mann–Whitney U test). A pathogenic or likely pathogenic variant was detected in 39 patients (19.9%), while 165 patients (80.9%) had no variants identified. Likely pathogenic and pathogenic variants were reevaluated according to the newest gene-specific guidelines: In five cases, variant classification was downgraded to variants of unknown clinical significance; thus, they were subsequently regrouped to negative cases. The final proportion of positive cases was 16.7%.

**Figure 2:**
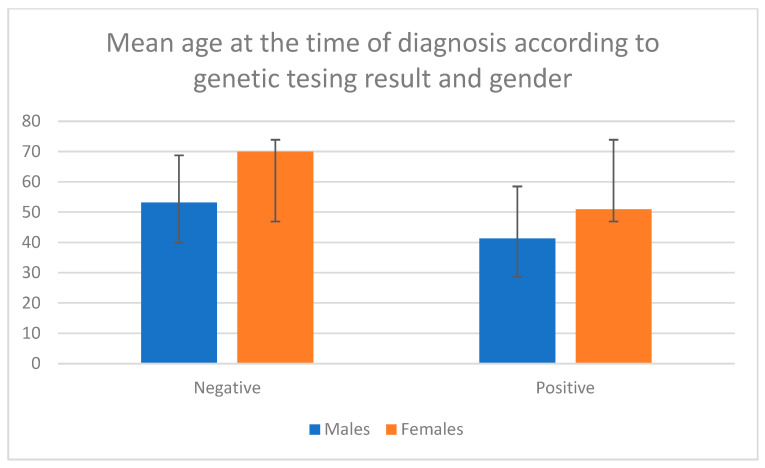
Mean age at the time of diagnosis according to genetic testing results and gender. Negative means no pathogenic variant was detected. Positive means a pathogenic variant was detected.

Patients with a detected variant were significantly younger at the time of diagnosis than those without a variant (median age 44 vs. 56 years, *p* < 0.001, Mann–Whitney U test). This trend was consistent across both genders: males with a pathogenic variant (n = 24) had a mean age at diagnosis of 41.33 ± 15.55 years compared to 53.81 ± 13.24 years in males without a variant, while females with a pathogenic variant (n = 10) had a mean age at diagnosis of 50.90 ± 17.13 years compared to 62.96 ± 12.55 years in those without a variant (Figure 2). A statistically significant difference was observed, with females being older in the group with no variant detected but not in the group with a pathogenic variant (*p* < 0.001, Mann–Whitney U test). The frequency of detected pathogenic variants did not differ significantly between males and females (Chi-square test, *p* = 0.892).

Patients with a pathogenic variant detected had, on average, greater MWT (20.61 ± 5.94 vs. 18.69 ± 4.47 mm; *p* = 0.053) but lower LVMi (106.53 ± 3 5.42 vs. 115.84 ± 37.01; *p* = 0.133), according to the Mann–Whitney U test.

The frequency of affected family members and the presence of symptoms at the time of testing did not differ significantly between patients with and without pathogenic variants. An overall positive family history was observed in 20.4% of analyzed cases: 28.1% of individuals with a pathogenic variant and 18.9% of those without one. Symptoms were present in 82% of the analyzed patients: in 81.2% of those with a pathogenic variant and in 82% of those without one. Interestingly, patients with a confirmed genetic cause had lower body mass index (BMI: 26.74 ± 5.07 vs. 31.31 ± 22.13, *p* = 0.004, Mann–Whitney U test). BMI was not correlated with either LVMi or MWT (Spearman’s correlation, *p* > 0.05).

Considering unfavorable outcomes of HCM, we tended to see more in the genotype-positive group, as more patients needed a reduction of the heart septal wall (17.6% vs. 9.4%), and more cases of cardioverter implantations (14.7% vs. 10.1%). Thus, differences were observed but were not statistically significant.

*MYBPC3* was the most frequently identified gene among patients with a detected causative variant (N = 20, 58.8%). Other affected genes were as follows: *MYH7* (N = 11, 32.4%), *PTPN11* (N = 2, 5.9%), and *TNNI3* (N = 1, 2.9%). All analyzed patients were affected by isolated HCM. We did not expect to find *PTPN11* gene pathogenic variants, as they usually cause Noonan or other related syndromes. At the first genetic consultation, no dysmorphic features or congenital anomalies were noted. Upon phenotype review, multiple lentigines were determined in two patents. After discussion with the medical team, LEOPARD syndrome (MIM 176876) was assigned in both cases. These patients had regular sun exposure in their professions, which likely masked suspicion of the syndrome earlier.

Patients with *MYBPC3* gene-causative variants were diagnosed with HCM earlier than those with *MYH7* (33.59 ± 15.68 vs. 43.10 ± 18.40). They also had greater MWT (23.08 ± 6.30 vs. 19.20 ± 6.09) and greater LVMi (115.24 ± 40.54 vs. 93.14 ± 20.69), although these differences were not statistically significant (Table 2). No apparent statistical differences (Chi square) were determined in the family history (5 vs. 3, *p* = 0.077), the presence of symptoms at testing (16 vs. 7, *p* = 0.713), or gender between patients (males 77.3% vs. 60.0%, *p* = 0.314) with causative variants in *MYBPC3* or *MYH7* genes.

All detected variants are presented in Table 3. Despite repeated variants, none of the patients were known to be related. Seven cases were found with *MYBPC3* variant c.1505G>A and four cases with *MYBPC3* variant c.3697C>T, while other variants were less frequent. When analyzing the distribution of detected variants, we noted that all *MYBPC3* variants affected exon 15–33, whereas *MYH7* variants were more widely dispersed. Nine variants of *MYH7* were in the myosin motor domain and three in the coiled coil domain. No significant differences in clinical parameters were found when comparing recurrent variants to others or comparing affected domains.

## 3. Discussion

We analyzed patients tested at the Hospital of Lithuanian University of Health Sciences Kauno Klinikos, a single center for genetic causes of HCM. We achieved a diagnostic yield of 16.7%, which is lower than in other studies [7,8,9,10]. If we exclude non-sarcomeric genes (like *PTPN11*), yield decreases to 15.7%. Based on recently published studies, there is a recent tendency to report lower diagnostic yields than previously thought. Comparison with these studies is challenging, as they differ in clinical selection criteria, tested gene panels, and variant interpretation guidelines. Some studies also included other cardiovascular genes, such as *SCN5A* and *RYR2*, whose variants do not necessarily explain the HCM phenotype [10]. In our study, we focused only on genes that are clearly related to HCM. Our lower diagnostic yield may also be related to overlapping phenotypes between HCM and other, more common cardiac disorders, such as severe arterial hypertension or valvular disease.

As expected, in our study, patients with an identified genetic cause of HCM were diagnosed earlier and had more pronounced MWT. This is in line with previously published studies, which have demonstrated that carriers of pathogenic variants in sarcomeric genes present with more severe disease and experience more major cardiac events [11]. However, multiple studies have shown that there is no clear correlation between the specific causative gene and the cardiac phenotype. We also noted that females in our study were diagnosed, on average, later with HCM compared to males. Terauchi Y. et al. also reported that females are usually diagnosed later but present with a more severe phenotype [12]. The same observation was made by Preveden A. et al. [13]. Looking globally, it is noted that males have a higher prevalence not only of HCM but also of myocarditis and ischemic heart disorders [14]. It is unknown whether male gender has biological predisposition regarding developing HCM or whether it is biased by different cardiac screening approaches or patients’ behavior towards seeking medical help [15]. These findings suggest that females might require a different clinical approach for early HCM detection.

Our results showed an association between pathogenic variants and patients’ BMI. This suggests that patients with higher BMI have a cardiac phenotype similar to that seen in genetically determined HCM. However, BMI was not associated with MWT, LVMi, symptoms, or other clinical parameters, indicating that this could be an incidental finding.

As mentioned, we have identified pathogenic variants in the *PTPN11* gene that are causative for LEOPARD syndrome. This syndrome usually presents with multiple lentigines, cardiac abnormalities, short stature, dysmorphic features, and other clinical signs [16]. Both of our patients carried variants affecting the same codon, 468, which has previously been reported in LEOPARD syndrome patients [17]. Based on the genetic test results and the presence of multiple lentigines, the diagnosis of this syndrome was established in our patients. Our gene panel included more genes than is usually recommended for isolated cases. According to the literature, the PTPN11 gene is typically excluded from panels [18,19,20]. However, considering that such syndromes can have variable expression and that cardinal features (e.g., dysmorphic traits) may diminish later in life, including syndromic HCM genes (phenocopies) in gene panels is valuable. This approach proved useful in our patients, who presented without short stature or dysmorphic features. It is also important to note that, in LEOPARD syndrome, HCM penetrance is approximately 80%, which is comparable to that observed for sarcomeric genes [21].

The most commonly affected genes in our study were *MYBPC3* and *MYH7*. Findings across multiple studies are similar [22]. In our study, we did not find any differences regarding age of onset, affected family members, MWT, or other parameters, suggesting limited impact of genotype on disease prognosis. Other studies have also reported no significant differences [23]. Patients with *MYBPC3* variants can be divided into two main groups: with truncating or with missense variants. As *MYBPC3* causes HCM through haploinsufficiency, we could expect a more severe phenotype with truncating variants. However, studies have shown no substantial impact on clinical presentation, likely due to the unclear pathomechanism of missense variants [11]. Similarly, our *MYBPC3*-positive patients did not demonstrate major phenotypic differences. The *MYBPC3* variants c.3467dup, c.1503C>G, c.2610dup, and c.1251del have not previously been described in the literature or clinical databases. They were classified as pathogenic due to their rarity in population databases and their predicted deleterious nature.

In our study, all *MYBPC3* variants were located in exons 15–33. Other studies have not reported clustering of truncating variants [21]. However, it was demonstrated that missense variants tend to affect the C3, C6, and C10 domains [21]. In our cases, *MYBPC3* variants affected the C3 and C8 domains compared to the sequence published by Carrier L et al. [24]. C3–C6 are the central domains participating in binding to other sarcomere proteins [25] and C8–10 anchor to the titin [26]. It is important to note that 32% of our patients with a positive *MYBPC3* finding harbored the p.Arg502Gln variant. In previously published studies, the most frequent variant is p.Arg502Trp, which accounts for approximately 1.5–3% of cases [27]. Our identified p.Arg502Gln variant is also relatively frequent and has previously been reported in cohorts from Poland [28], Jordan [29], and China [30].

*MYH7* causes HCM through a different mechanism: a dominant-negative of poisonous polypeptide. Incorporation of the modified protein into the sarcomere leads to functional changes. There are many different variants in the MYH7 gene that can lead to HCM through various protein changes, in most cases leading to an increased number of myosin heads available to interact with actin [31]. For example, the variant p.Gly256Glu in the literature is described as more benign than others, with a lower likelihood of sudden cardiac death. Nevertheless, it is also described in pediatric patients with expressed phenotype [32]. Our patient with this variant presented with disease at 53 years old, with no affected family members (possibly due to reduced penetrance). Therefore, an individual *MYH7* genotype may not provide an accurate prognosis. It is speculated that variants in myosin motor regions can be severe [33]. This is supported by variants reported in databases, as more pathogen variants are within codons 167–931 [34]. Our three cases are outside this region (2x p.Thr1377Met; p.Arg1420Gln), in the coiled coil domain. However, these cases also did not differ significantly from others. These variants are not novel and well described in the literature.

Our study is limited by the small sample size, and thus may not reach the necessary statistical power. Not all possible confounding factors were analyzed, as there are many clinical situations that can cause left ventricular hypertrophy, such as intensive training, untreated long-going arterial hypertension, etc. Also, not all patients selected for genetic testing met the required criteria for HCM diagnosis.

## 4. Materials and Methods

This study was conducted between 2019 and 2024 in the Hospital of Lithuanian University of Health Sciences Kauno Klinikos, Genetics and Molecular Medicine department. The study was approved by the Kaunas Regional Biomedical Research Ethics Committee (No. BE-2-63). Data were collected from electronic health records and from patients who were consulted due to diagnosed or clinically suspected HCM (confirmed by heart ultrasound and/or heart MRI). Inclusion criteria: patients aged 18 years or older at the time of genetic consultation, echocardiography or cardiac MRI performed, and HCM diagnosed or suspected by a multidisciplinary medical team consisting of a clinical geneticist and cardiologists. The cohort consisted of patients who had had an HCM diagnosis for years, as well as those who were newly diagnosed. Data on symptoms, age at onset, laboratory and instrumental findings, complications such as atrial/ventricular fibrillation, treatments such as cardioverter/pacemaker implantation, and septal wall reduction (myectomy or alcohol ablation) were collected. Maximum wall thickness (MWT) of the left ventricle of the heart, left ventricular mass indexed LVMi, and heart ejection fraction (EF) were determined by the first available heart MRI scan. The MRI scans were performed on a 3T magnetic resonance imaging scanner with an 18-channel cardiac coil (MAGNETOM Skyra, Siemens Healthcare, Erlangen, Germany). Images were acquired during an expiratory breath hold with ECG gating. In case MRI was not performed (or no data were accessible), heart ultrasound parameters were used. Two-dimensional (2D) transthoracic echocardiography (TTE) at rest was performed by an experienced echocardiographer using a diagnostic ultrasound system (EPIQ 7, Phillips Ultrasound, Inc., Bothell, WA, USA). All measurements were performed according to the existing guidelines. Family history was considered positive if the first-degree relatives had sudden cardiac death before the age of 60 years, or if there were known cases of HCM in the family.

Patients’ DNA was extracted from peripheral blood samples using the DNA extraction system QIA symphony (QIAGEN, Hilden, Germany) with the reagent kit DNA midi Kit or with QIcube (QIAGEN, Hilden, Germany), with the reagent QIAmp DNA blood Mini Kit. DNA purity was measured with Qubit (Thermo Fisher Scientific, Waltham, MA, USA), using dsDNA HS Assay kit reagents. All patients underwent genetic testing using the Sistemas Genómicos (Valencia, Spain) Cardio-GeneSGKit library preparation kit and the Illumina NextSeq 550 system (San Diego, CA, USA). The library kit gene list changed during the period of analysis, but without compromising the core analysis genes. The gene lists are in the Supplementary Materials (List S1—for older panel, List S2—newest panel). All laboratory procedures were carried out according to the manufacturers’ manuals. The enrichment targeted all the genes associated with hereditary cardiovascular disorders, with an average sequencing depth of 200×. Samples that did not reach the target average depth were re-sequenced until the required depth was achieved. Most genes were entirely covered. Only coding exons and 20 bp of intron–exon boundaries were analyzed and reported. Sequencing fragment alignment, quality evaluation, variant calling, and gene variant interpretation were performed using the Sistemas Genomics (provided with the library kit) online platform *GeneSystems*. Low-quality variants (coverage < 10×, allele balance < 20%, Phred < 20) were removed. We selected only those genes for analysis that showed evidence of causing HCM by PanelApp: *ACTC1*, *ACTN2*, *ALPK3*, *CACNA1C*, *CSRP3*, *FHL1*, *FHOD3*, *FLNC*, *GLA*, *LAMP2*, *MYBPC3*, *MYH7*, *MYL2*, *MYL3*, *PLN*, *PRKAG2*, *TBX20*, *TNNC1*, *TNNI3*, *TNNT2*, *TPM1*, *TRIM63*, and *TTR* [18]. Firstly, variants were classified according to the ACMG/AMP 2015 guidelines [35]. Because the study is retrospective, variant interpretation criteria varied between periods, so we reinterpreted variants according to the newest gene specific recommendations from ClinGen [36,37]. Variants of unknown clinical significance were not included in this analysis; however, clinically relevant variants were reported to the patients.

Statistical data were collected in a Microsoft Office Excel spreadsheet. Statistical analysis was performed with IBM SPSS 26.0 software. All data were checked for normal distribution with the Kolmogorov–Smirnov test. As data were not normally distributed, non-parametric tests were used as appropriate based on data type: Chi square, Mann–Whitney U test, and Spearmen test.

## 5. Conclusions

Genetic testing in Lithuanian HCM patients revealed that *MYBPC3* and *MYH7* are the most frequently affected genes, with several novel variants identified. Patients with pathogenic variants were diagnosed earlier and exhibited a more severe phenotype, highlighting the value of genetic screening in HCM management.

## Figures and Tables

**Table 1:** Main clinical parameters studied.

Parameter		All	Genotype Positive	Genotype Negative	*p*-Value
Total, n (%)		204	34 (16.7%)	17 (83.3%)	
	males	144	24 (16.9%)	118 (83.1%)	
	female	62	10 (16.10%)	52 (83.9)	
Average age at testing		57.71 ± 13.05	49.05 ± 15.12	56.39 ± 11.98	<0.001
	males	54.74 ± 12.98	46.48 ± 15.10	56.39 ± 11.90	0.003
	female	63.87 ± 13.04	55.23 ± 13.98	65.53 ± 12.31	0.024
Average age at diagnosis			44.15 ± 16.38	56.61 ± 13.70	<0.001
	males		41.33 ± 15.55	53.81 ± 13.24	<0.001
	female		50.90 ± 17.13	62.96 ± 12.66	0.032
Affected family members (n = 201)		41 (20.4%)	9(28.1%)	32 (18.9%)	0.237
Symptoms present at testing (n = 200)		164 (82%)	27 (81.8%)	137 (82%)	0.976
MWT (n = 204), mm		19.00 ± 4.78	20.61 ± 5.94	18.69 ± 4.47	0.053
LVMi (n = 196), g/m^2^		114.41 ± 36.89	106.53 ± 35.42	115.84 ± 37.01	0.133
EF (n = 204), %		63.52 ± 1271	63.84 ± 12.63	63.46 ± 12.76	0.966
BNP (n = 160), ng/L		222.16 ± 345.13	250.26 ± 304.81	216.45 ± 353.53	0.139
Septal reduction performed (n = 203)		22 (10.7%)	6 (17.6%)	16 (9.4%)	0.62
ICD (n = 203)		22(10.8%)	5 (14.7%)	17 (10.1%)	0.426
Pacemaker		18 (8.9%)	3 (8.8%)	15 (8.9%)	0.992
AF (n = 203)		44 (21.7%)	6 (17.6%)	38 (22.5%)	0.532
VF/VT (n = 203)		35 (16.7%)	8 (23.5%)	27 (15.4%)	0.273

MWT—maximum wall thickness, LVMi—left ventricular mass indexed, EF—heart ejection fraction, BNP—brain natriuretic peptide, ICD—implanted cardioverted defibrillator, AF—atrial fibrillation, VF/VT—ventricular fibrillation/tachycardia. For continuous data, the Mann–Whitney U test was used; for categorical data, the Chi square test was used.

**Table 2:** Patient data distribution with respect to *MYBPC3* and *MYH7* variants.

Gene	Variant Affect	Age at Diagnosis (Years)	MWT Mean ± SD mm	LVMi Mean ± SD g/m^2^
*MYBPC3*	All variants	39.59 ± 15.68	23.08 ± 6.30	115.24 ± 40.54
	Truncating	41.75 ± 16.43	21.50 ± 5.47	102.08 ± 27.55
	Missense	37.00 ± 15.17	24.98 ± 7.00	135.92 ± 50.74
*MYH7*	All variants	43.10 ± 18.4	19.2 ± 6.09	93.14 ± 20.69

MWT—maximum wall thickness in mm; LVMi—left ventricular mass indexed in g/m^2^.

**Table 3:** Variants detected in our cohort.

Gene	Coding Variant	Protein Variant	Variant Effect	Affected Exon	Pathogenicity Classification Criteria	Classification	Novel	Gender	Age at Diagnosis	Family History	Present Symptoms at Time of Testing	Maximum Wall Thickness
*MYBPC3*	c.1251del	p.Lys418SerfsTer4	Frameshift	15/35	PVS1vstr, PM2s	LP	Yes	M	46	0	1	28
*MYBPC3*	c.1503C>G	p.Tyr501Ter	Nonsense	17/35	PVS1vstr, PM2s	LP	yes	M	37	1	1	19
*MYBPC3*	c.1504C>T	p.Arg502Trp	Missense	17/35	PM1m, PS4str, PP1str	P	No	M	17	0	NA	16
*MYBPC3*	c.1505G>A	p.Arg502Gln	Missense	17/35	PM1m, PS4str, PP1s	LP	No	M	38	1	1	20
*MYBPC3*	c.1505G>A	p.Arg502Gln	Missense	17/35	PM1m, PS4str, PP1s	LP	No	M	35	1	1	28
*MYBPC3*	c.1505G>A	p.Arg502Gln	Missense	17/35	PM1m, PS4str, PP1s	LP	No	F	44	0	1	29
*MYBPC3*	c.1505G>A	p.Arg502Gln	Missense	17/35	PM1m, PS4str, PP1s	LP	No	M	22	0	1	34
*MYBPC3*	c.1505G>A	p.Arg502Gln	Missense	17/35	PM1m, PS4str, PP1s	LP	No	F	34	0	1	24
*MYBPC3*	c.1505G>A	p.Arg502Gln	Missense	17/35	PM1m, PS4str, PP1s	LP	No	M	53	0	1	27
*MYBPC3*	c.1505G>A	p.Arg502Gln	Missense	17/35	PM1m, PS4str, PP1s	LP	No	M	55	0	1	23
*MYBPC3*	c.2373dup	p.Trp792fs	Frameshift	24/35	PVS1vstr, PS4str	P	No	F	42	0	1	26
*MYBPC3*	c.2610dup	p.Ser871fs	Frameshift	26/35	PVS1vstr	LP	yes	M	35	0	1	33
*MYBPC3*	c.2827C>T	p.Arg943Ter	Nonsense	27/35	PVS1vstr, PS4m	P	No	M	72	NA	1	22
*MYBPC3*	c.3064C>T	p.Arg1022Cys	Missense	29/35	PP3s	VUS	No	M	56	1	1	14
*MYBPC3*	c.3467dup	p.Pro1157fs	Frameshift	31/35	PVS1vstr, PM2s, PS4str	P	Yes	M	12	0	0	23
*MYBPC3*	c.3467dup	p.Pro1157fs	Frameshift	31/35	PVS1vstr, PM2s, PS4str	P	Yes	M	19	1	0	15
*MYBPC3*	c.3467dupA	p.Pro1157fs	Frameshift	31/35	PVS1vstr, PM2s, PS4str	P	No	M	39	0	0	22
*MYBPC3*	c.3697C>T	p.Gln1233Ter	Nonsense	33/35	PVS1vstr, PS4str	P	No	M	36	0	1	21
*MYBPC3*	c.3697C>T	p.Gln1233Ter	Nonsense	33/35	PVS1vstr, PS4str	P	No	M	53	NA	1	17
*MYBPC3*	c.3697C>T	p.Gln1233Ter	Nonsense	33/35	PVS1vstr, PS4str	P	No	F	60	0	0	17
*MYBPC3*	c.3697C>T	p.Gln1233Ter	Nonsense	33/35	PVS1vstr, PS4str	P	No	M	50	0	1	15
*MYBPC3*	c.3763G>A	p.Ala1255Thr	Missense	33/35	PM1m, PP3s, PS4s	VUS	No	F	16	0	0	35
*MYH7*	c.1207C>T	p.Arg403Trp	Missense	13/40	PM1m, PP3s, PP1str, PS4str	P	No	M	31	0	1	24
*MYH7*	c.1324C>T	p.Arg442Cys	Missense	14/40	PP3s, PM1m, PP1m, PS4str	LP	No	M	25	0	1	22
*MYH7*	c.1548C>A	p.Asp516Glu	Missense	15/40	PM2s, PM1m, PP3s	VUS	No	M	22	1	0	31
*MYH7*	c.1988G>A	p.Arg663His	Missense	18/40	PP3s, PM1m, PP1str, PS4str	P	No	M	40	0	1	20
*MYH7*	c.1988G>A	p.Arg663His	Missense	18/40	PP3s, PM1m, PP1str, PS4str	P	No	F	67	1	1	20
*MYH7*	c.2167C>T	p.Arg723Cys	Missense	20/40	PM1m, PS2str, PP1m, PS4m	P	No	F	80	0	1	17
*MYH7*	c.4130C>T	p.Thr1377Met	Missense	30/40	PP3s, PM2s, PS4str, PP1str	P	No	F	43	1	1	9
*MYH7*	c.4130C>T	p.Thr1377Met	Missense	30/40	PP3s, PM2s, PS4str, PP1str	P	No	M	46	0	1	20
*MYH7*	c.4259G>A	p.Arg1420Gln	Missense	31/40	PP3s, PM5s, PS4str	LP	No	M	57	0	1	15
*MYH7*	c.746G>A	p.Arg249Gln	Missense	9/40	PM2s, PP3s, PM1m, PS4str, PS3m	P	No	F	23	1	0	18
*MYH7*	c.767G>A	p.Gly256Glu	Missense	9/40	PM1m, PP3s, PM2s, PS4str, PP1m	P	No	M	53	0	1	14
*MYH7*	c.958G>A	p.Val320Met	Missense	11/40	PP3s, PM1m, PM2s, PS4str	LP	No	F	51	0	0	22
*MYL3*	c.382G>T	p.Gly128Cys	Missense	4/7	PM2s, PP3s	VUS	No	F	29	0	1	16
*MYL3*	c.382G>T	p.Gly128Cys	Missense	4/7	PM2s, PP3s	VUS	No	M	44	0	0	18
*PTPN11*	c.1402A>C	p.Thr468Pro	Missense	12/16	PP2s, PM5m, PM2s, PP3s, PS4m, PS2m, PS3s	P	No	M	54	1	1	8
*PTPN11*	c.1403C>T	p.Thr468Met	Missense	12/16	PP2s, PP3s, PS3m, PS4str, PM6m	P	No	M	67	1	1	18
*TNNI3*	c.433C>T	p.Arg145Trp	Missense	7/8	PM1m, PP3s, PS3m, PS4str, PP1s	P	No	F	65	0	1	15

The “Novel” column indicates whether the variant was previously described in the literature or databases. The “Gender” column shows the biological gender of the patients (F—biological females, M—biological males). The “Family History” column indicates whether there was a first-degree family member with HCM or another early-onset (<60) cardiac disorder (0—no affected family members, 1—at least one affected family member). The “Present Symptoms” column indicates whether the patients had symptoms related to heart disorder (chest pain, shortness of breath, palpitations). “Pathogenicity Classification Criteria” shows abbreviations according to ACMG variant interpretation guidelines. P—pathogenic, LP—likely pathogenic, VUS—variance of unknown clinical significance. NA—not available.

## Data Availability

The original contributions presented in this study are included in the article/Supplementary Materials. Further inquiries can be directed to the corresponding author.

## Supplementary Materials

The following supporting information can be downloaded at: https://www.mdpi.com/article/10.3390/ijms27010221/s1.

## Abbreviations

The following abbreviations are used in this manuscript:

AF	Atrial fibrillation
BNP	Brain natriuretic peptide
HCM	Hypertrophic cardiomyopathy
ICD	Implantable cardioverter defibrillator
MWT	Heart maximum wall thickness
LVMi	left ventricular mass indexed
VF/VT	Ventricular fibrillation/tachycardia
