# Supplementary material for: Hypertrophic Cardiomyopathy Genotype–Phenotype Analysis in Lithuanian Single-Center Cohort
Source: Int J Mol Sci. 2025 Dec 25;27(1):221. doi: 10.3390/ijms27010221 (PMC12785989; doi:10.3390/ijms27010221)
Supplement: Supplementary file 1 [file ijms-27-00221-s001.zip › ijms-4000226-supplementary.pdf]

List S1. Gene panel used for the samples until 2020 May. ABCA1, ABCC6, ABCC9, ABCG5, ABCG8, ACTA1, ACTA2, ACTC1, ACTN2, ADRB1, ADRB2, ADRB3, AGL, AGRN, AKAP9, ANGPTL3, ANK2, ANKRD1, ANO5, APOA1, APOB, APOE, ATP2A1, ATP7A, BAG3, BIN1, BMPR2, BRAF, CACNA1B, CACNA1C, CACNA1D, CACNA2D1, CACNB2, CALR3, CAPN3, CASQ2, CAV3, CBL, CFC1, CFL2, CHKB, CLCN1, CNTN1, COL3A1, COL4A1, COL6A1, COL6A2, COL6A3, COL9A3, CRYAB, CSRP3, CTF1, CYP7A1, DAG1, DES, DMD, DNM2, DOK7, DPP6, DSC2, DSG2, DSP, DTNA, DYSF, EFEMP2, ELN, EMD, ENPP1, EYA4, FBLN5, FBN1, FBN2, FHL1, FHL2, FKRP, FKTN, FLNC, FXN, G6PC3, GAA, GATA4, GATA6, GDF1, GJA1, GJA5, GLA, GLMN, GNE, GPD1L, GSN, HCN1, HCN4, HRAS, HSPG2, IGHMBP2, ILK, ISCU, ITGA7, JAG1, JPH2, JUP, KBTBD13, KCNA5, KCND3, KCNE1, KCNE1L, KCNE2, KCNE3, KCNE4, KCNH2, KCNJ11, KCNJ12, KCNJ2, KCNJ3, KCNJ5, KCNJ8, KCNQ1, KCNQ2, KRAS, LAMA2, LAMA4, LAMP2, LARGE, LDB3, LDLR, LDLRAP1, LMNA, LPL, LRP6, MAP2K1, MAP2K2, MATR3, MEF2A, MTM1, MYBPC3, MYH11, MYH2, MYH6, MYH7, MYL2, MYL3, MYLK, MYLK2, MYOT, MYOZ2, MYPN, NEB, NEBL, NEXN, NKX2-3, NKX2-5, NKX2-6, NOTCH1, NPPA, NRAS, OPA1, PABPN1, PCSK9, PDLIM3, PKP2, PKP4, PLEC, PLEKHG5, PLN, PLOD3, PNN, POMGNT1, POMT1, POMT2, PRKAG2, PSEN1, PSEN2, PSMB8, PTPN11, PTRF, RAF1, RANGRF, RASA1, RBM10, RBM20, RPSA, RYR1, RYR2, SCN10A, SCN1B, SCN2B, SCN3B, SCN4B, SCN5A, SCNN1B, SCNN1G, SDHA, SEPN1, SGCA, SGCB, SGCD, SGCG, SHOC2, SLC25A4, SLC2A10, SMAD3, SMN1, SMN2, SNTA1, SOS1, SPRED1, SYNE1, SYNE2, TAZ, TBX1, TBX20, TCAP, TGFB3, TGFBR1, TGFBR2, TGFBR3, TLL1, TMEM43, TMPO, TNNC1, TNNI3, TNNT1, TNNT2, TPM1, TPM2, TPM3, TRIM32, TRPM4, TRPV4, TTN, TTR, UBA1, VAPB, VCL, VCP, VRK1, ZFPM2, ZNF469

List S2. Gene panel used after 2020 May. A2ML1, ABCC6, ABCC9, ACTA2, ACTC1, ACTN2, ACVR2B, ACVRL1, AGL, AKAP9, ALG10, ANK2, ANKRD1, BAG3, BMPR2, BRAF, CACNA1B, CACNA1C, CACNA1D, CACNA2D1, CACNB2, CALM1, CALM2, CALM3, CALR3, CASQ2, CAV1, CAV3, CBL, CBS, CFC1, CHD7, CHST14, CITED2, COL1A1, COL1A2, COL3A1, COL4A1, COL5A1, COL5A2, CRELD1, CRYAB, CSRP3, CTNNA3, DES, DMD, DPP6, DSC2, DSG2, DSP, DTNA, EFEMP2, EHMT1, EIF2AK4, ELN, EMD, ENG, ENPP1, EVC, EYA4, FBLN5, FBN1, FBN2, FHL1, FHL2, FHOD3, FKBP14, FKRP, FKTN, FLNA, FLNC, FOXC1, FOXF1, FXN, G6PC3, GAA, GATA4, GATA6, GATAD1, GBE1, GDF1, GDF2, GJA1, GJA5, GLA, GLMN, GNAI2, GPD1L, GUCY1A3, GYG1, GYS1, HAND2, HCN4, HFE, HRAS, HTRA1, ILK, JAG1, JPH2, JUP, KCNA5, KCND2, KCND3, KCNE1, KCNE2, KCNE3, KCNE4, KCNE5, KCNH2, KCNJ2, KCNJ5, KCNJ8, KCNK17, KCNK3, KCNQ1, KRAS, KRIT1, LAMA4, LAMP2, LDB3, LDLR, LMNA, LOX, LRP6, LZTR1, MAP2K1, MAP2K2, MEF2A, MFAP5, MIB1, MMP21, MYBPC3, MYH11, MYH6, MYH7, MYL2, MYL3, MYLK, MYLK2, MYOT, MYOZ2, MYPN, NEBL, NEXN, NF1, NKX2-3, NKX2-5, NKX2-6, NODAL, NOTCH1, NOTCH2, NOTCH3, NPPA, NRAS, NUP155, OBSCN, PDLIM3, PKP2, PKP4, PLEC, PLN, PLOD1, PRDM16, PRKAG2, PRKG1, PROC, PSEN1, PSEN2, PTPN11, RAF1, RANGRF, RASA1, RASA2, RBM10, RBM20, RIT1, RNF213, RPSA, RRAS, RYR1, RYR2, SCN1B, SCN2B, SCN3B, SCN4B, SCN5A, SDHA, SGCD, SHOC2, SLC25A4, SLC2A10, SLMAP, SMAD3, SMAD4, SMAD6, SMAD9, SNTA1, SOS1, SOS2, SPRED1, SYNE1, SYNE2, TAB2, TAZ, TBX1, TBX20, TBX4, TBX5, TCAP, TEK, TFAP2B, TGFB2, TGFB3, TGFBR1, TGFBR2, TLL1, TMEM43, TMPO, TNNC1, TNNI3, TNNI3K, TNNT2, TNXB, TPM1, TRDN, TRPM4, TTN, TTR, VCL, XK, ZFPM2, ZIC3, ZNF469
